# Using the MoBI motion capture system to rapidly and accurately localize EEG electrodes in anatomic space

**DOI:** 10.1111/ejn.15019

**Published:** 2021-02-21

**Authors:** Kevin A. Mazurek, Eleni Patelaki, John J. Foxe, Edward G. Freedman

**Affiliations:** 1 The Cognitive Neurophysiology Laboratory, The Del Monte Institute for Neuroscience, Department of Neuroscience, University of Rochester School of Medicine and Dentistry, Rochester, NY, USA; 2 Department of Biomedical Engineering, University of Rochester, Rochester, NY, USA

**Keywords:** EEG, electrode locations, motion capture

## Abstract

During mobile brain/body imaging (MoBI) experiments, electroencephalography and motion capture systems are used in concert to record high temporal resolution neural activity and movement kinematics while participants perform demanding perceptual and cognitive tasks in a naturalistic environment. A typical MoBI setup involves positioning multi-channel electrode caps based on anatomical fiducials as well as experimenter and participant intuition regarding the scalp midpoint location (i.e., Cz). Researchers often use the “template” electrode locations provided by the manufacturer, however, the “actual” electrode locations can vary based on each participant’s head morphology. Accounting for differences in head morphologies could provide more accurate clinical diagnostic information when using MoBI to identify neurological deficits in patients with motor, sensory, or cognitive impairments. Here, we asked whether the existing motion capture system used in a MoBI setup could be easily adapted to improve spatial localization of electrodes across participants without requiring additional or specialized equipment that might impede clinical adoption. Using standard electrode configurations, infrared markers were placed on a subset of electrodes and anatomical fiducials, and the remaining electrode locations were estimated using spherical or ellipsoid models. We identified differences in event-related potentials between “template” and “actual” electrode locations during a Go/No-Go task (*p*< 9.8e–5) and an object-manipulation task (*p*< 9.8e–5). Thus, the motion capture system already used in MoBI experiments can be effectively deployed to accurately register and quantify the neural activity. Improving the spatial localization without needing specialized hardware or additional setup time to the workflow has important real-world implications for translating MoBI to clinical environments.

## 1 | INTRODUCTION

Mobile brain/body imaging (MoBI) experiments involve recording neural activity from an electroencephalography (EEG) system and movement kinematics from a motion capture system (Makeig et al., 2009). Such experiments allow for identifying changes in neural activity as additional motor processes (e.g., walking) are added to the existing experimental paradigm (De Sanctis et al., 2012, 2014; Gramann et al., 2011; Jungnickel & Gramann, 2016; Malcolm et al., 2015, 2018, 2019; Parada & Rossi, 2020). Neural source localization requires accurately identifying the scalp locations of EEG electrodes. Source localization techniques are useful for solving the inverse problem in order to predict the loci of neural activity in the brain that resulted in the recorded activity on the scalp (Grech et al., 2008; Scherg & Von Cramon, 1986). One approach is to assume that every participant has a similar head configuration and use a template to estimate where in the brain neural activity likely originated. Determining the spatial location of the electrodes relative to anatomical fiducials on the head can provide more accurate source localization results to compare task-related responses during MoBI experiments across participants (Shirazi & Huang, 2019b).

Recent studies have demonstrated that MoBI has the potential to provide vital information to clinicians to understand the changes in neural mechanisms in different patient cohorts (e.g., multiple sclerosis, Alzheimer’s disease, or Parkinson’s disease). One potential limitation to applying MoBI to different clinical populations is ensuring the electrode spatial positions are consistent across participants. Taking into account differences in patients’ head morphologies could provide more accurate diagnostic information when for clinicians using MoBI. There is a need to have an approach to accurately spatially localize electrodes for each patient, however, the ease of use for such an approach should not add significant time or resources to the clinical workflow. There are several electrode localizing systems and techniques that have been developed to solve this problem (Chen et al., 2019; Clausner et al., 2017; Cline et al., 2018; Song et al., 2018; Taberna, Marino, et al., 2019). Polhemus (Polhemus LTD) has a system that requires using a wand and pressing down on the tops of each electrode to register the spatial location (Gevins et al., 1990). Another novel system uses a 3-D scanner to reconstruct the electrode locations (Homolle & Oostenveld, 2019). Toolboxes have been developed to process and localize the 3-D output from these systems (Taberna, Guarnieri, et al., 2019; Taberna, Marino, et al., 2019). There are several other methods for spatially localizing the EEG electrodes using virtual reality systems (Cline et al., 2018), augmented reality systems (Song et al., 2018), photogrammetry (Clausner et al., 2017), and time of flight camera systems (Chen et al., 2019), however, each of these approaches requires an additional hardware system that could impede the translation of MoBI to clinical use.

Taking advantage of the existing motion capture system already used in MoBI experiments to localize EEG electrodes could provide an approach for clinical use that would not involve needing an additional hardware system. Previous studies have demonstrated that motion capture systems are capable of spatially localizing EEG electrodes by placing infrared (IR) markers on the top of each electrode (Reis & Lochmann, 2015; Shirazi & Huang, 2019b). Reis and Lochmann demonstrated that EEG electrodes were spatially localized with comparable accuracy to using CT imaging (Reis & Lochmann, 2015). Their system did require a custom-modified electrode cap (Brain Products GmbH) to prevent artifacts from the overlapping markers and cables (Reis et al., 2014). Being able to use the existing motion capture system with standard electrode caps and IR markers that are already used for a typical MoBI experiment could provide an approach that is easy to when translating to clinical use.

In the work presented here, we wanted to determine if a subset of IR markers could be used to estimate the electrode spatial locations. Using as few as eight IR motion tracking markers, we estimated the remaining markers as healthy participants performed a MoBI experiment. As a sub-analysis to the study, we wanted to compare the sensitivity of the event-related potentials (ERPs) based on the identified electrode errors similar to other studies (Akalin Acar & Makeig, 2013; Beltrachini et al., 2011; Dalal et al., 2014; He & Musha, 1989; Shirazi & Huang, 2019a, 2019b). Previous MoBI studies have compared the timing and amplitude of specific ERP components between healthy individuals and patient cohorts to identify changes in neural activity related to cognitive-motor impairments (De Sanctis et al., 2020; Malcolm et al., 2015, 2018). Spatial variability of electrode locations could affect the signal-to-noise of the ERP components when performing such group average analyses. Determining how electrode spatial locations errors affected the ERPs in the MoBI experiments proposed here could help determine whether clinicians should even localize each patients’ electrode positions or rely on consistently placing the electrodes to then use the template locations provided by the manufacturer.

## 2 | MATERIALS AND METHODS

### 2.1 | Human subjects

10 participants aged 20–72 (four female) participated in the study. Five performed a MoBI go/no-go task similar to De Sanctis and colleagues (De Sanctis et al., 2014) and five performed an object-manipulation task similar to Mazurek and colleagues (Mazurek et al., 2020). All participants had normal or corrected-to-normal vision, and reported no history of psychiatric, neurological, or musculoskeletal disorders. The Institutional Review Board of the University of Rochester approved the experimental procedures, and all participants provided their written informed consent. All procedures were compliant with the principles laid out in the Declaration of Helsinki for the responsible conduct of research.

### 2.2 | Experimental setup—Capturing the EEG electrodes locations using motion capture

**FIGURE 1:**
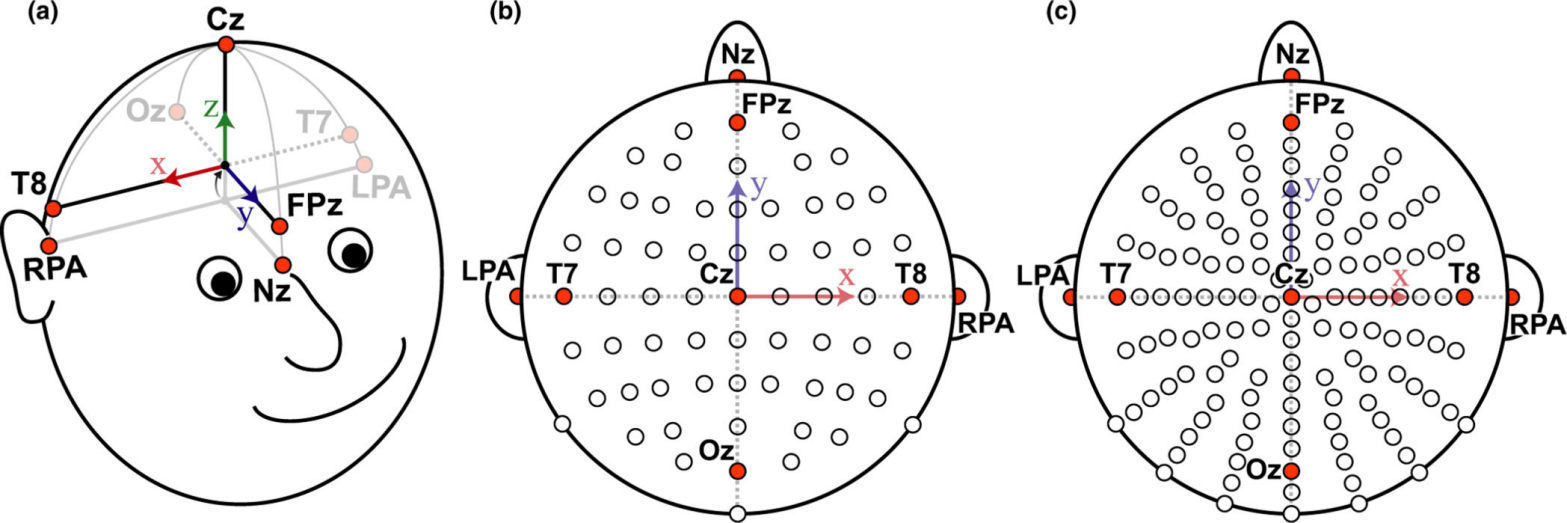
Schematic of the local coordinate space for spatially localizing the electrodes. (a) The coordinate axis is defined such that the*x*axis points to the right ear (aligned with electrode T8), the*y*-axis points to the nose (aligned with FPz), and the*z*-axis points to the top of the head (aligned with electrode Cz). The origin was initially defined by the midpoint between the fiducial markers LPA and RPA and the intersection with the Nasion (Nz). This origin was then translated along the*z*-axis to the average of the z-components of the FPz, Oz, T7, and T8 markers. (b) Top view of the 64-electrode configuration (10–20 layout) with the electrodes that had IR markers highlighted in orange. (c) Same as B for the 128-electrode configuration. LPA, left preauricular; RPA, right preauricular

A motion capture system (Optitrack Inc.) was used to record the EEG electrode locations from either a 64- or 128-electrode configuration, industry standard electrode cap without any modifications (BioSemi Inc.). The 64-electrode configuration is in the traditional 10–20 layout (Jasper, 1958; Sharbrough et al., 1991), the 128-electrode configuration has electrode positions radially equidistant from electrode Cz. Electrode caps were placed on each participant’s head relative to the anatomical fiducials as described by the manufacturer specifications (BioSemi Inc.). Participants then provided feedback about where they thought the “top” of their head was to help confirm placement of electrode Cz. Using the 64-electrode configuration, participants performed a MoBI Go/No-Go task in which a 16 camera motion capture system was used in a ~75 m^2^ space (Prime 41 cameras; Optitrack Inc.). Using the 128-electrode configuration, participants performed an object-manipulation task in which a 13 camera motion capture system was used in a ~7.5 m^2^ electrically isolated booth (Prime 13W cameras; Optitrack Inc.). Electrode locations were recorded from IR markers placed on specific electrodes. Markers were also placed on three anatomical landmarks (Nasion [Nz], left preauricular [LPA], and right preauricular [RPA]) which were used to align the electrode locations for each participant (Figure 1a). For both the 64- and 128-electrode configurations, infrared reflective markers were placed on the top of the following electrodes or at the following fiducial locations (Figure 1b,c): Cz, Oz, FPz, T7, T8, Nz, LPA, and RPA (Sharbrough et al., 1991). Markers used were 6.4 mm diameter M3 reflective markers (part number MKR064M3; Optitrack Inc.) on a 15 mm × 5 mm M3 marker base (part number MB15W05HM3; Optitrack Inc.), and the exposure level in the Motive software was set to 250 μs for all cameras and the measurement error after calibration was <0.5 mm. The offset from the center of the M3 marker to the bottom of the marker base was 10 mm and the height of the electrode was measured to be 11 mm, so an offset of 21 mm was corrected relative to the measured local coordinate space (defined in Section 2.3). IR marker locations were recorded using the native Motive Software (Optitrack Inc.) and using lab streaming layer (LSL; Kothe, 2014) to synchronize with the experimental data. Marker locations were then imported to Matlab (Mathworks Inc.) for subsequent analysis.

### 2.3 | Analysis—Defining the local coordinate space for the EEG electrode locations

The EEG electrode locations were determined from the IR markers using the following procedure. The coordinate axis was defined such that the *x* direction pointed to the right ear, the *y* direction pointed toward the nose, and the *z* direction pointed towards the top of the head (Figure 1). The electrode locations were pre-processed to align to this coordinate space using markers placed on the fiducial (LPA, RPA, Nz) as a reference. First, we translated all the rigid body markers to use the midpoint of the LPA and RPA markers as the new origin. Next, we rotated all the markers such that the Nz marker was aligned with the *y*-axis with coordinates (0, *y*_Nasion_, 0). Markers were then rotated about the *y*-axis to align the LPA and RPA markers with the *x*-axis. Fiducial markers were placed assuming the vectors from the origin to LPA (or RPA) and the origin to the Nz were orthogonal. We performed a small correctional rotation to the LPA and RPA marker locations (median rotation of 2.1 degrees [25th and 75th percentiles: 1.2° and 3.9°, respectively]) to ensure the vectors were orthogonal (and thus ensure the coordinate axes were orthogonal). Finally, to align with the coordinate space of the template electrode locations provided by the manufacturer, we shifted the *z*-component of the origin from the midpoint of the LPA and RPA fiducials to the midpoint of the T7, T8, Oz, and FPz markers to estimate the *z*-position centered in the middle of the head. We made this assumption to more accurately compare between the actual electrode locations and the template locations. These resulting local coordinate axes were then equivalent to the template electrode coordinate space relative to the anatomical fiducials.

### 2.4 | Analysis—Estimating electrode locations using a spherical or ellipsoid head model

**FIGURE 2:**
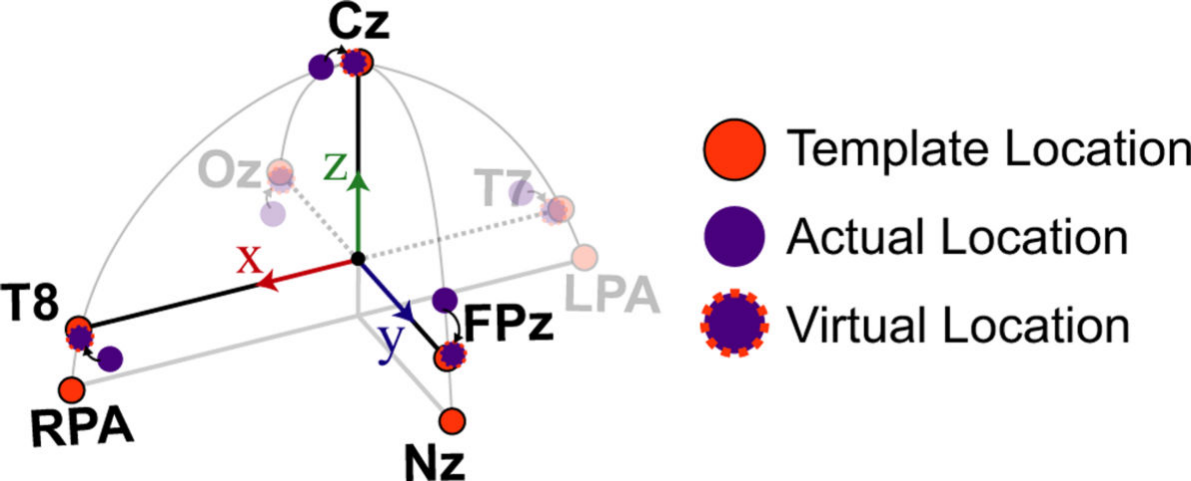
Schematic of how the virtual electrode locations were estimated from the actual electrode locations to better compare with the template locations. The template locations (orange) assume that the T7/T8 electrodes are parallel with LPA/RPA, and the Cz is aligned with the z-axis. Fixing the anatomical fiducials (LPA, RPA, and Nz), the other electrodes were rotated to align Cz with the z-axis and to make the vector between T7 and T8 parallel with the vector defined by LPA and RPA. Template electrode locations are depicted in orange, actual electrode locations are depicted in purple, and the virtual electrodes are depicted in purple with an orange dashed outline. Coordinate axes the same as Figure 1a. LPA, left preauricular; Nz, Nasion; RPA, right preauricular

Once the local coordinate space was established for each participant, we then created spherical and ellipsoid models of the head to estimate the remaining electrodes and compare against the template electrode locations. To create these models, we needed to rotate the actual electrode locations to “virtual” electrode locations that allowed for estimating the radii of the head and were more consistent with the template electrode locations relative to the LPA, RPA, and Nz fiducials (schematic shown in Figure 2). Fixing the fiducial locations, we first rotated the actual electrode coordinates such that the vector between T7 and T8 and LPA and RPA were parallel. Next, we rotated all the electrode markers about the *x*-axis to align the Cz marker with the *z*-axis. After performing these two rotations, the resulting electrode locations were defined as the “virtual” electrode locations and used to define the radii of the head. For the spherical model, the distance between the origin and the “virtual” Cz marker was used as the estimated head radius (*r*). The azimuth (*φ*) and inclination (*θ*) angles were obtained from the manufacturer specifications (BioSemi Inc.). The cartesian coordinates were then computed using the following equations:

x=r⋅sinθ⋅cosφ,

y=r⋅sinθ⋅sinφ,

z=r⋅cosθ.

For the ellipsoid model, we estimated the three radii for each axis and the same azimuth and inclination angles were used as in the spherical model. The distance from the origin to the “virtual” T8 marker was used as the radius in the *x* dimension (*r*_*x*_); the distance from the origin to the “virtual” FPz marker was used as the radius in the *y* dimension (*r*_*y*_); the distance from the origin to the “virtual” Cz marker was used as the radius in the z dimension (*r*_*z*_). These radii were used to estimate the remaining electrode locations using the following equations:

$$x=r_{x}\cdot sin\theta \cdot cos\varphi ,$$

$$y=r_{y}\cdot sin\theta \cdot sin\varphi ,$$

$$z=r_{z}\cdot cos\theta .$$

### 2.5 | Behavioral task—Go/No-Go task

Participants performed a Go/No-Go MoBI task similar to De Sanctis et al. (2014) during which 64 electrodes were recorded in a standard 10–20 configuration (Jasper, 1958; Sharbrough et al., 1991). Participants are presented with images taken from the International Affective Picture System (IAPS) database (Lang et al., 1997). The IAPS database contains images of varied emotional valence, however, for this study we collapsed trials of all emotional valences. Images were presented every 1,000 ms during which the image is displayed for 60 ms. Participants were instructed to press a button if the presented image was different from the preceding image (“Go” Trial) or to withhold pressing the button if the presented image was the same as the preceding image (“No-Go” Trial). Participants performed blocks of 240 trials in which 209 were Go Trials and 31 were No-Go Trials (87% vs. 13%). No-Go trials were randomly distributed in each block. Blocks of trials were performed either sitting or walking on a treadmill. An experimental session consisted of 16 blocks: one training block at the beginning, seven sitting blocks, seven walking blocks, and one task-free block (walking on the treadmill without a task). The order of sitting and walking blocks was pseudorandomized; no more than three consecutive walking blocks occurred to prevent tiring the participants.

### 2.6 | Behavioral task—Object-manipulation task

Participants performed an object-manipulation task similar to Mazurek et al. (2020) during which 128 electrodes were recorded using a standard configuration with electrodes radially equidistant from electrode Cz. Participants were instructed either with visual or auditory stimuli about what object pair to contact. Stimuli lasted for 300 ms and instructed the participant to (a) use a hammer to hit a peg; (b) put a cookie in a cookie jar; or (c) use a drill to tighten a screw. Participants held a large home button for 2,000 to 2,500 ms before receiving the instruction. Instruction delivery was defined as when the stimuli were delivered and logged in Presentation. Movement onset was defined as when participants released the home button which triggered a Transistor–transistor logic (TTL) pulse logged by Presentation. Object contact was defined as when participants picked up the target object and opened a magnetic switch embedded in each object. Objects were placed in three locations 15 cm radially from the home button. Participants performed blocks of 12 trials that were presented pseudorandomly (three objects, two sensory stimuli) with objects at each location. Each experiment involved participants using their left hand and right hand in separate blocks of trials.

For both the Go/No-Go task and the object-manipulation task, stimulus triggers from Presentation (Neurobehavioral Systems Inc.), behavioral responses from the buttons and switches, motion tracking data, and EEG data were time-synchronized using LSL. Motion capture data were recorded using custom software written to rebroadcast the data from the Motive software to the LSL labrecorder. EEG Data were recorded from available LSL streaming plugins for the BioSemi system. Behavioral event markers were recorded using built-in the LSL functionality in the Presentation software. All behavioral, EEG, and motion kinematic data analyses were performed using custom MATLAB scripts (MathWorks) and/or functions from EEGLAB (Delorme & Makeig, 2004).

### 2.7 | Analysis—Comparing ERPs between actual and template electrode locations

Electroencephalography data were recorded using a BioSemi Active Two System (BioSemi Inc.) using either 64 or 128 electrode configurations. Neural activity was digitized at 2,048 Hz, re-referenced to the common average, downsampled to 512 Hz, and bandpass filtered using a zero-phase Chebyshev Type II filter (1–55 Hz). Next, “bad” channels were detected based on kurtosis, probability, and spectrum of the recorded data, setting the threshold to 10% of the normalized measure value, as well as covariance, with the threshold set to ± 3 *SD*s. These “bad” channels were removed and interpolated based on neighboring channels. Independent component analysis was used to remove artifacts (e.g., eyeblinks, muscle activity, ground noise, etc.) using the ICLabel algorithm (Pion-Tonachini et al., 2019). ICs labeled at artifacts were removed and the remaining ICs were back projected to the sensor space.

The resulting neural activity was then split into temporal epochs beginning 200 ms before and extending until 800 ms after stimulus onset for each trial. Epochs were baselined to zero the amplitude from −100 to 0 ms relative to stimulus onset. Epochs with a maximum voltage greater than ±75 μV or exceeded 10 standard deviations in terms of kurtosis and probability were excluded from further analysis. For the Go/No-Go task, ERPs were measured by averaging epochs for the four experimental conditions: Go Trials (sitting), No-Go Trials (sitting), Go Trials (walking), No-Go Trials (walking). For the object-manipulation task, ERPs were measured by averaging epochs for the three experimental conditions: instruction sensory modality (visual/auditory), target object (drill, hammer, cookie), and object location (left, center, right). For this study, only ERPs for the Go Trials (sitting) for the Go/No-Go task and all visually instructed trials for the object-manipulation task were used to demonstrate differences in ERPs based on how electrodes were spatially localized. Comparison with ERPs during other conditions was beyond the scope of this study.

Event-related potentials were measured at the actual location of the eight IR markers and then spatially interpolated to the template location (defined by the manufacturer). ERPs were spatially interpolated using the scattered Interpolant function in Matlab (Mathworks Inc.) based on the estimated ellipsoid model of the head. ERPs from the actual and template locations were then statistically compared using a paired-sample *t* test (function *t* test) in Matlab at each time point of the trial and Bonferroni corrected for the number of time points (1 s trial, 512 Hz, Bonferroni corrected *α* = 0.05/512≈9.8e–5).

### 2.8 | Statistical analyses

All statistical analyses were performed using Matlab (Mathworks, Inc.). The distances between electrode locations estimated using spherical or ellipsoid template models and the actual electrode locations from the IR markers were compared using a Wilcoxon signed-rank test (*p*< .05). The ERPs were compared using a paired-sample *t* test and the significance level was Bonferroni corrected for the number of time stamps compared (0.05/512≈9.8e–5).

## 3 | RESULTS

Using the motion capture system already integrated in a MoBI experiment, we recorded the spatial locations from five electrodes (Cz, Fpz, Oz, T7, and T8) and three fiducial locations (LPA, RPA, and Nz) to estimate the remaining electrode positions. For each participant, we estimated “virtual” electrode locations that aligned Cz on the *z*-axis and T7 and T8 along the *x*-axis in order to estimate the radii of the head. We compared the distance between the actual and virtual electrode locations for the five electrodes where IR markers were placed to determine how far off the actual electrode placement was from aligning these axes. For the 64-electrode configuration, the median distance between the actual and virtual electrodes was 14.9 mm (25th and 75th percentiles: 10.4 and 21.7 mm, respectively). For the 128-electrode configuration, the median distance was 10.7 mm (4.3 and 16.7 mm).

**FIGURE 3:**
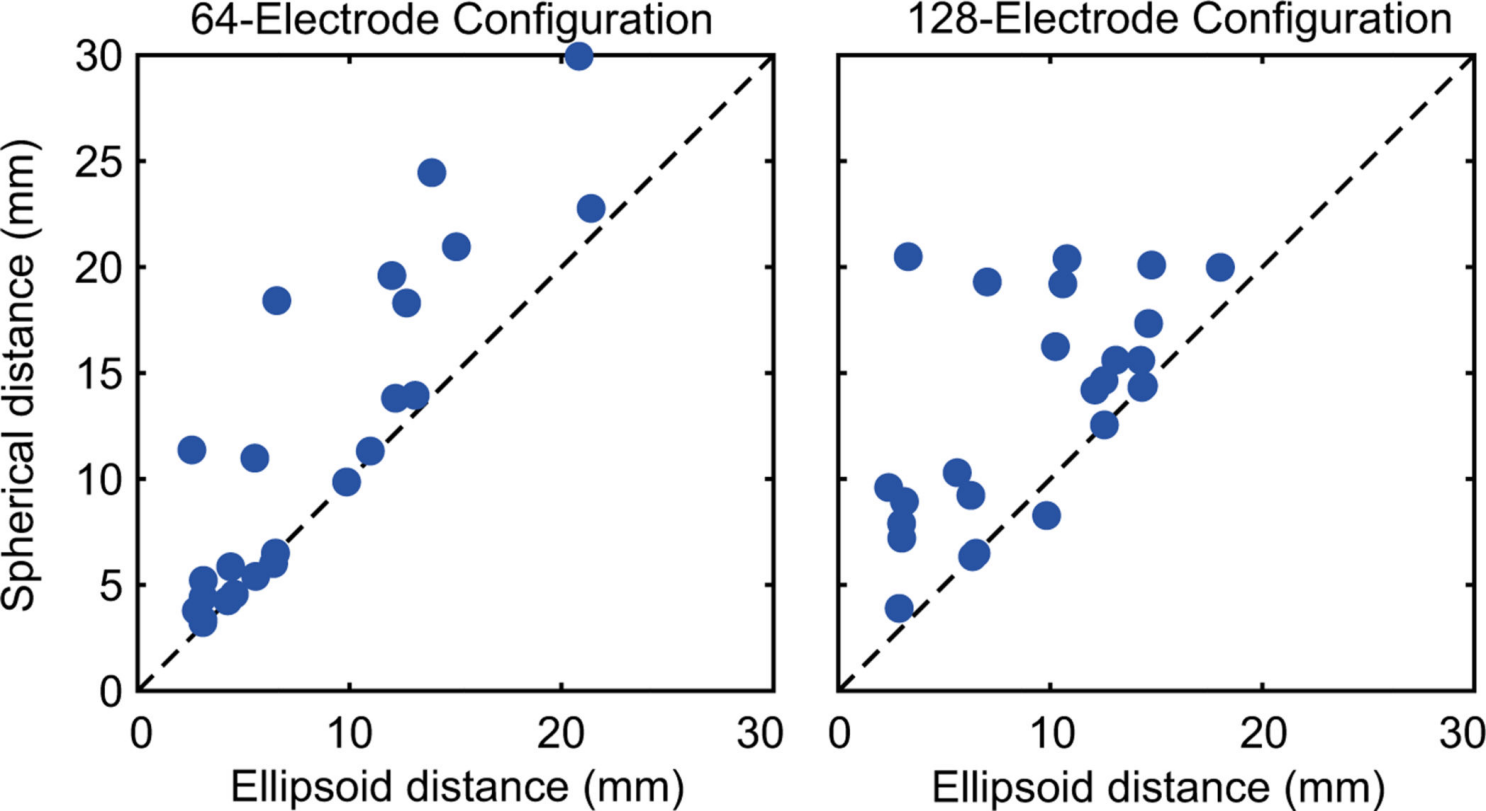
Comparisons of the distances from the electrode locations estimated from the spherical or ellipsoid models to the template locations for the electrodes on which IR markers were placed. Datapoints are shown for the (a) 64-electrode configuration or (b) 128-electrode configuration. Each datapoint represents the Euclidean distance from the template location and the ellipsoid estimate (*x*-axis) or the spherical estimate (*y*-axis). Dashed line represents the unity line (*y* = *x*). Data points above the unity line indicate the spherical distance is greater than ellipsoid distance (and vice versa). For both configurations, the ellipsoid distance was less than the spherical distance (Wilcoxon signed-rank tests)

After determining the virtual electrode locations, spherical and ellipsoid models of each participant’s head were estimated based on the head radii. As a measure of goodness of fit, Figure 3 depicts a scatter plot comparing the Euclidean distances between the virtual electrode locations and the spherical model estimates and the ellipsoidal model estimates. For the 64-electrode configuration, distances for the ellipsoid model (6.4 [4.0 12.3] mm, median [25th, 75th percentile]) were closer to the virtual marker locations compared to the spherical model (9.9 [4.6 18.3] mm], Wilcoxon signrank test, *p* = 2.5e–4). Similarly for the 128-electrode configuration, ellipsoid model distances (10.2 [5.0 13.4] mm) were less than the spherical model distances (14.3 [8.8 17.8] mm, *p* = 1.4e–4).

**FIGURE 4:**
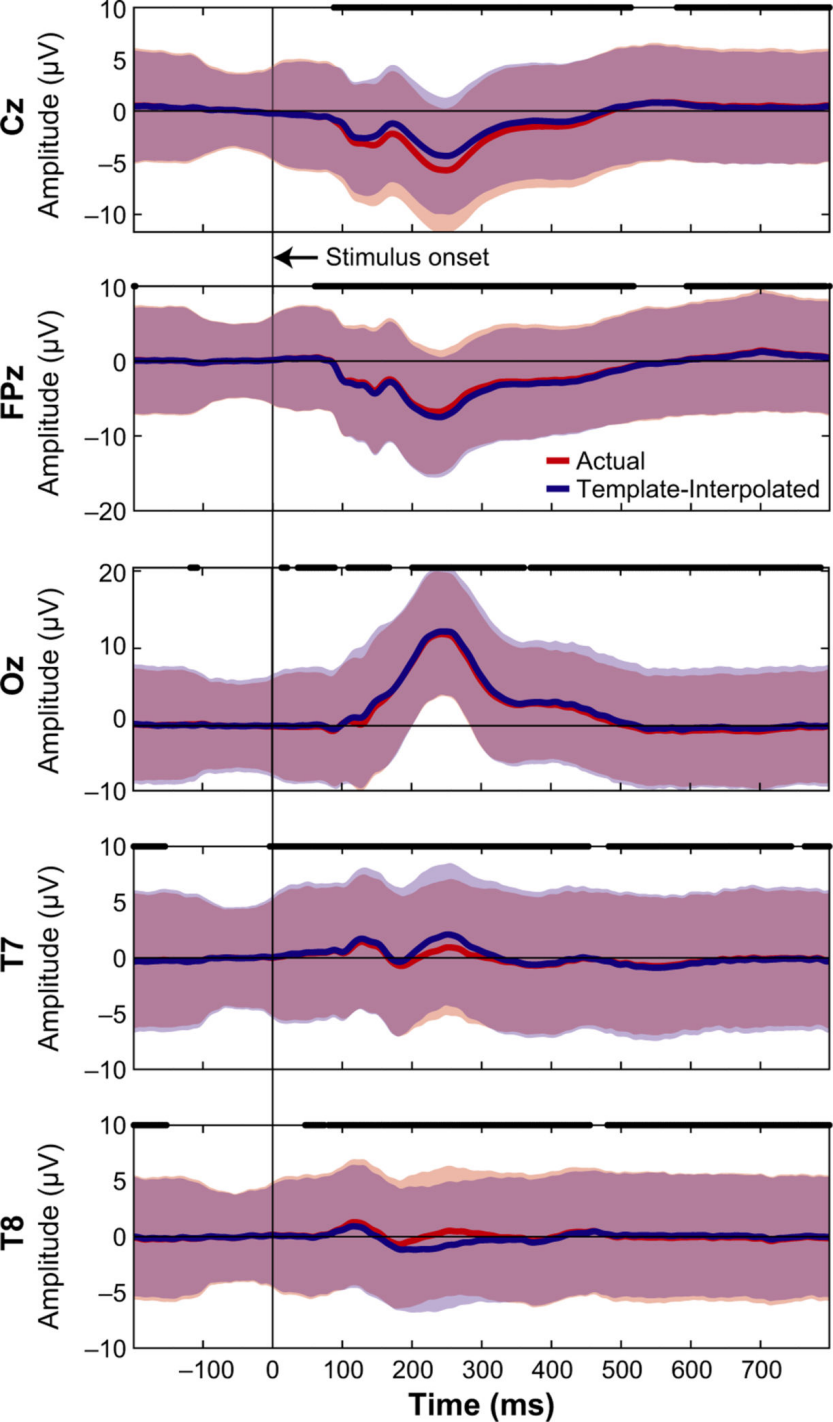
Event-related potentials Go Trials (sitting) during a Go/No-Go task. The five electrodes locations were depicted by averaging across participants based on where the marker was (“actual” location in red) and interpolated to the virtual electrode location using an ellipsoid model of each participant (“templateinterpolated” in blue). A black line at time 0 indicates the stimulus onset. Thick black lines correspond to when the actual and template-interpolated ERPs were significantly different (*t*test, Bonferroni corrected for the number of time stamps,*α* = 0.05/512≈9.8e–5). Shaded regions indicate ± 2*SD*s from the mean. ERP, event-related potential

**FIGURE 5:**
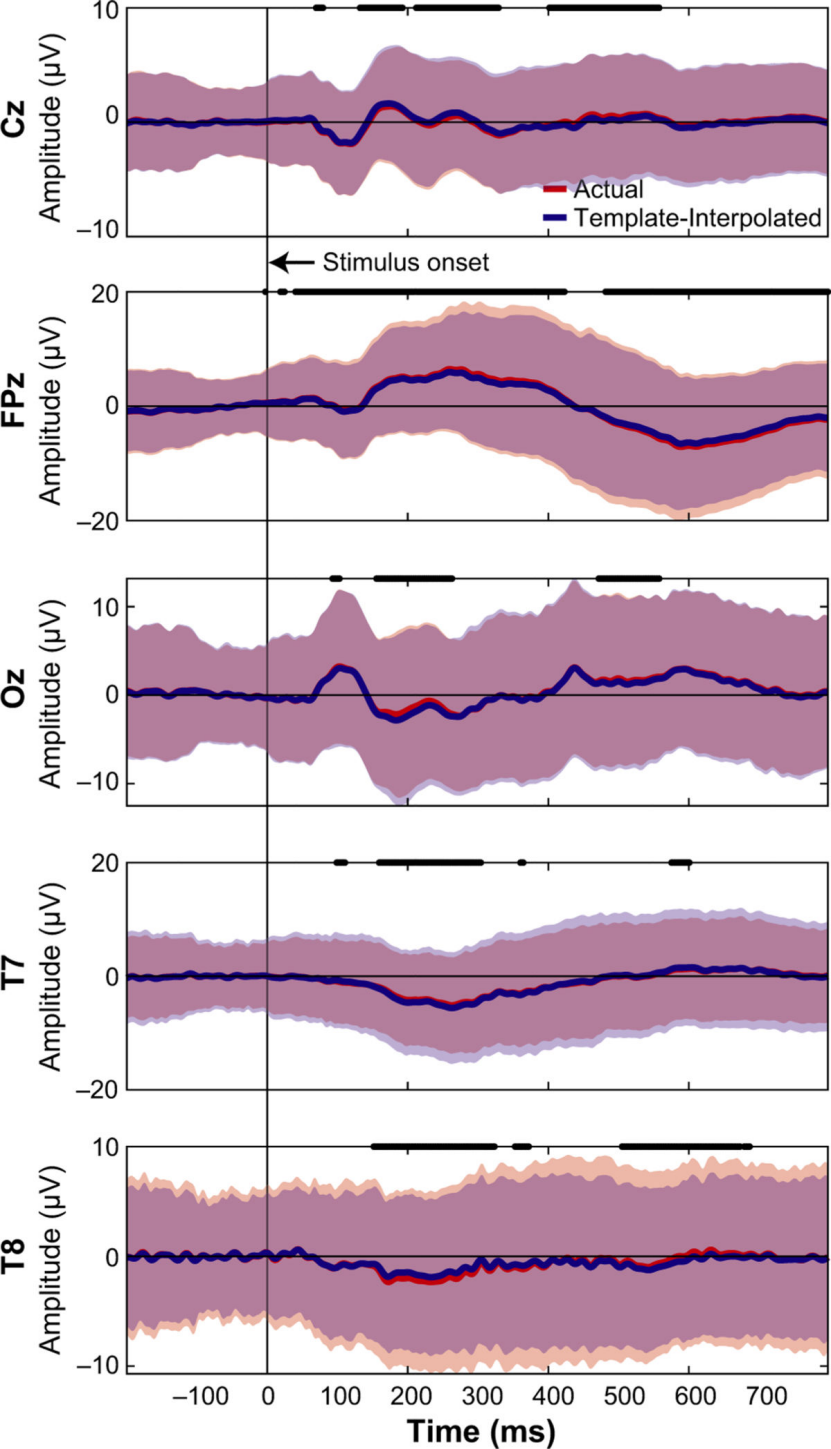
Comparing ERPs between actual and template-interpolated locations from the object manipulation task using 128 electrodes. Conventions same as Figure 4. ERP, event-related potential

We next wanted to determine how much the neural activity changed after adjusting for electrode location errors between the actual IR measured electrodes and the template locations, similar to previous studies (Akalin Acar & Makeig, 2013; Beltrachini et al., 2011; Dalal et al., 2014; He & Musha, 1989; Shirazi & Huang, 2019a, 2019b). As a sub-analysis, we looked at the ERPs from the 64 channel recordings collected during the MoBI Go/No-go task and the ERPs from the 128 channel recordings during the object-manipulation task to determine whether the neural responses differed at the actual versus the template electrode locations. We used the virtual electrode locations (Figure 2) to spatially interpolate the ERPs from the actual locations as an estimate of the template-interpolated ERPs. In Figure 4, we plotted ERPs from Go Trials (sitting) for the five electrodes where we also placed the IR markers (Cz, FPz, Oz, T7, and T8). For locations. Figure 5 depicts the ERPs aligned on visual instruction onset for the five electrodes on which IR markers were placed. Again, each of the electrodes had significant differences between the ERP at the actual location versus the virtual electrode location (*p*< 9.8e–5). Thus, shifts in electrode placement have an effect on how the ERPs are presented. Depending on the analyses being performed during a MoBI experiment, these differences in electrode location could attenuate or amplify findings and affect interpretation each marker, red traces correspond to ERPs using the actual electrode locations and blue traces correspond to ERPs after using an ellipsoid model to estimate the recorded EEG from each participant at the template location (interpolated). For each of the electrodes, significant differences were detected between the actual and interpolated ERPs (two-sided paired *t* test, *p*< 9.8e–5, Bonferroni corrected for the 512 timesteps). Similarly for the object-manipulation task, the ERPs were compared between where the electrodes were placed and where they should have been based on the virtual electrode of the results which is important for clinical use.

## 4 | DISCUSSION

Here, we demonstrate an approach for using MoBI technologies to estimate the spatial locations of EEG electrodes using only eight IR markers (five EEG electrodes and three anatomical fiducials). As a motion capture system is already a key component of a MoBI experiment, using it to spatially localize the electrodes does not require additional hardware and adds minimal time to the experiment. Consistent placement of the EEG cap is crucial for identifying neural changes in a clinical MoBI experiments. In the experiments described here, we used a subset of IR markers to improve the spatial localization of the electrodes in a manner that would be quick to accomplish for clinical MoBI applications. Other studies have demonstrated that electrode could be accurately localized using motion capture systems (Reis & Lochmann, 2015; Shirazi & Huang, 2019b). In the experiments conducted here, we spatially localized the electrodes using only a subset of markers to correct for any errors/variability in the initial electrode placement without needing specialized electrode caps or placing markers on every electrode. For MoBI experiments involving patients with motor impairments, neurological disorders, or cognitive deficits, relying on participant identification of the “top” of the head as feedback could result in much greater variability in electrode placement even if the initial placement was accurate relative to anatomical fiducials. Using a subset of IR markers allowed us to correct for these differences relative to the template electrode positions and did not add a significant amount of time to the experimental workflow. Such an approach is advantageous for having a consistent, reproducible method for placing EEG electrodes for participants of all abilities that would be useful for clinical MoBI applications.

The proposed approach is not without its limitations. Placing IR markers on every electrode would provide a more complete characterization about the spatial locations. However, this can become time consuming as the number of electrodes increases, and artifacts can be introduced as more cables interfere with detecting the electrode positions or as the density of electrodes approaches the motion capture system’s measurement error. We reduced the amount of time by only recording five EEG electrode locations and estimating the remaining based on the 10/20 azimuth and elevation coordinates. This approach provides a fairly accurate estimate when using an ellipsoid model of the head, however, even this is not a fully accurate representation of the head morphology. Using other localization systems such as virtual reality, augmented reality, or time of flight camera systems (Chen et al., 2019; Clausner et al., 2017; Cline et al., 2018; Song et al., 2018; Taberna, et al., 2019) or putting IR markers on all the electrodes could overcome the small inaccuracies in our approach (Reis & Lochmann, 2015; Shirazi & Huang, 2019b). Several studies have analyzed how errors in electrode locations affect neural source estimations from EEG (Akalin Acar & Makeig, 2013; Beltrachini et al., 2011; Dalal et al., 2014; He & Musha, 1989; Shirazi & Huang, 2019a, 2019b), thus to incorporate any of these systems for adoption into clinical applications would need to take into account the trade-off of improved neural localization errors with added time to the clinical workflow.

Another approach that could be taken to improve the consistency of cap placement across participants is to use the motion capture system to align the EEG cap to the fiducials in real-time based on the small subset of IR markers used. By visualizing the electrode locations during cap placement using the motion capture system, information about the angles between the fiducials and where the top of the cap should be could be corrected in real-time. Such an approach could improve the reproducibility and consistency in which electrode caps are placed across participants and provide a more quantitative measure of how each cap is placed, however, would also need to be developed to not add significant time to the workflow for clinical MoBI applications. Consistent electrode placement and localization will be vital when performing source localization to estimate the origin(s) of the observed neural activity during future clinical and non-clinical MoBI experiments. As more MoBI studies are conducted which recruit patients of with different motor, sensory, or cognitive impairments (De Sanctis et al., 2020; Malcolm et al., 2015), having an automated approach to either correct for differences in electrode placement post hoc or improve the consistency of how the electrodes are placed in real-time will improve the reliability of comparing neural responses across participants.

## Abbreviations:

EEG: electroencephalography
ERP: event-related potential
IR: infrared
MoBI: mobile brain/body imaging
Mocap: motion capture

## References

[R1] Akalin Acar Z, & Makeig S. (2013). Effects of forward model errors on EEG source localization. Brain Topography, 26, 378–396. 10.1007/s10548-012-0274-6 23355112 PMC3683142

[R2] Beltrachini L, von Ellenrieder N, & Muravchik CH (2011). General bounds for electrode mislocation on the EEG inverse problem. Computer Methods and Programs in Biomedicine, 103, 1–9. 10.1016/j.cmpb.2010.05.008 20599288

[R3] Chen S, He Y, Qiu H, Yan X, & Zhao M. (2019). Spatial localization of EEG electrodes in a TOF+CCD camera system. Front Neuroinform, 13, 21. 10.3389/fninf.2019.00021 31024285 PMC6465776

[R4] Clausner T, Dalal SS, & Crespo-Garcia M. (2017). Photogrammetry-based head digitization for rapid and accurate localization of EEG electrodes and MEG fiducial markers using a single digital SLR camera. Frontiers in Neuroscience, 11, 264. 10.3389/fnins.2017.00264 28559791 PMC5432580

[R5] Cline CC, Coogan C, & He B. (2018). EEG electrode digitization with commercial virtual reality hardware. PLoS One, 13, e0207516. 10.1371/journal.pone.0207516 PMC6248988 30462691

[R6] Dalal SS, Rampp S, Willomitzer F, & Ettl S. (2014). Consequences of EEG electrode position error on ultimate beamformer source reconstruction performance. Frontiers in Neuroscience, 8, 42. 10.3389/fnins.2014.00042 24653671 PMC3949288

[R7] De Sanctis P, Butler JS, Green JM, Snyder AC, & Foxe JJ (2012). Mobile brain/body imaging (MoBI): High-density electrical mapping of inhibitory processes during walking. Proceedings of the Annual International Conference of the IEEE Engineering in Medicine and Biology Society, 2012, 1542–1545. 10.1109/embc.2012.6346236 23366197

[R8] De Sanctis P, Butler JS, Malcolm BR, & Foxe JJ (2014). Recalibration of inhibitory control systems during walking-related dual-task interference: A mobile brain-body imaging (MOBI) study. NeuroImage, 94, 55–64. 10.1016/j.neuroimage.2014.03.016 24642283 PMC4209901

[R9] De Sanctis P, Malcolm BR, Mabie PC, Francisco AA, Mowrey WB, Joshi S, Molholm S, & Foxe JJ (2020). Mobile brain/body imaging of cognitive-motor impairment in multiple sclerosis: Deriving EEG-based neuro-markers during a dual-task walking study. Clinical Neurophysiology, 131, 1119–1128. 10.1016/j.clinph.2020.01.024 32200093 PMC7196176

[R10] Delorme A, & Makeig S. (2004). EEGLAB: An open source toolbox for analysis of single-trial EEG dynamics including independent component analysis. Journal of Neuroscience Methods, 134, 9–21. 10.1016/j.jneumeth.2003.10.009 15102499

[R11] Gevins A, Brickett P, Costales B, Le J, & Reutter B. (1990). Beyond topographic mapping: Towards functional-anatomical imaging with 124-channel EEGs and 3-D MRIs. Brain Topography, 3, 53–64. 10.1007/BF01128862 2094314

[R12] Gramann K, Gwin JT, Ferris DP, Oie K, Jung TP, Lin CT, Liao LD, & Makeig S. (2011). Cognition in action: Imaging brain/body dynamics in mobile humans. Reviews in the Neurosciences, 22, 593–608. 10.1515/RNS.2011.047 22070621

[R13] Grech R, Cassar T, Muscat J, Camilleri KP, Fabri SG, Zervakis M, Xanthopoulos P, Sakkalis V, & Vanrumste B. (2008). Review on solving the inverse problem in EEG source analysis. Journal of NeuroEngineering and Rehabilitation, 5, 25. 10.1186/1743-0003-5-25 18990257 PMC2605581

[R14] He B, & Musha T. (1989). Effects of cavities on EEG dipole localization and their relations with surface electrode positions. International Journal of Bio-Medical Computing, 24, 269–282. 10.1016/0020-7101(89)90022-6 2606568

[R15] Homolle S, & Oostenveld R. (2019). Using a structured-light 3D scanner to improve EEG source modeling with more accurate electrode positions. Journal of Neuroscience Methods, 326, 108378. 10.1016/j.jneumeth.2019.108378 31376413

[R16] Jasper HH (1958). The ten-twenty electrode system of the International Federation. Electroencephalography and Clinical Neurophysiology, 10, 370–375. 10590970

[R17] Jungnickel E, & Gramann K. (2016). Mobile brain/body imaging (MoBI) of physical interaction with dynamically moving objects. Frontiers in Human Neuroscience, 10, 306. 10.3389/fnhum.2016.00306 27445747 PMC4921999

[R18] Kothe C. (2014) Lab streaming layer (LSL). Retrieved from https://github.com/sccn/labstreaminglayer

[R19] Lang PJ, Bradley MM, & Cuthbert BN (1997). International affective picture system (IAPS): Technical manual and affective ratings. NIMH Center for the Study of Emotion and Attention, 1, 39–58.

[R20] Makeig S, Gramann K, Jung TP, Sejnowski TJ, & Poizner H. (2009). Linking brain, mind and behavior. International Journal of Psychophysiology, 73, 95–100. 10.1016/j.ijpsycho.2008.11.008 19414039 PMC2796545

[R21] Malcolm BR, Foxe JJ, Butler JS, & De Sanctis P. (2015). The aging brain shows less flexible reallocation of cognitive resources during dual-task walking: A mobile brain/body imaging (MoBI) study. NeuroImage, 117, 230–242. 10.1016/j.neuroimage.2015.05.028 25988225 PMC5080979

[R22] Malcolm BR, Foxe JJ, Butler JS, Molholm S, & De Sanctis P. (2018). Cognitive load reduces the effects of optic flow on gait and electrocortical dynamics during treadmill walking. Journal of Neurophysiology, 120, 2246–2259. 10.1152/jn.00079.2018 30067106 PMC6295527

[R23] Malcolm BR, Foxe JJ, Butler JS, Mowrey WB, Molholm S, & De Sanctis P. (2019). Long-term test-retest reliability of event-related potential (ERP) recordings during treadmill walking using the mobile brain/body imaging (MoBI) approach. Brain Research, 1716, 62–69. 10.1016/j.brainres.2017.05.021 28532853 PMC7209996

[R24] Mazurek KA, Richardson D, Abraham N, Foxe JJ, & Freedman EG (2020). Utilizing high-density electroencephalography and motion capture technology to characterize sensorimotor integration while performing complex actions. IEEE Transactions on Neural Systems and Rehabilitation Engineering, 28, 287–296. 10.1109/TNSRE.2019.2941574 31567095 PMC7021210

[R25] Parada FJ, & Rossi A. (2020). Perfect timing: Mobile brain/body imaging scaffolds the 4E-cognition research program. European Journal of Neuroscience. 10.1111/ejn.14783 32422692

[R26] Pion-Tonachini L, Kreutz-Delgado K, & Makeig S. (2019). ICLabel: An automated electroencephalographic independent component classifier, dataset, and website. NeuroImage, 198, 181–197. 10.1016/j.neuroimage.2019.05.026 31103785 PMC6592775

[R27] Reis PM, Hebenstreit F, Gabsteiger F, von Tscharner V, & Lochmann M. (2014). Methodological aspects of EEG and body dynamics measurements during motion. Frontiers in Human Neuroscience, 8, 156. 10.3389/fnhum.2014.00156 24715858 PMC3970018

[R28] Reis PM, & Lochmann M. (2015). Using a motion capture system for spatial localization of EEG electrodes. Frontiers in Neuroscience, 9, 130. 10.3389/fnins.2015.00130 25941468 PMC4403350

[R29] Scherg M, & Von Cramon D. (1986). Evoked dipole source potentials of the human auditory cortex. Electroencephalography and Clinical Neurophysiology, 65, 344–360. 10.1016/0168-5597(86)90014-6 2427326

[R30] Sharbrough F, Chatrian G-E, Lesser RP, Luders H, Nuwer M, & Picton TW (1991). American Electroencephalographic Society guidelines for standard electrode position nomenclature. Journal of Clinical Neurophysiology, 8, 200–202. 10.1097/00004691-199104000-00007 2050819

[R31] Shirazi SY, & Huang HJ (2019a). Influence of mismarking fiducial locations on EEG source estimation (pp. 377–380). 2019 9th International IEEE/EMBS Conference on Neural Engineering (NER). IEEE.

[R32] Shirazi SY, & Huang HJ (2019b). More reliable EEG electrode digitizing methods can reduce source estimation uncertainty, but current methods already accurately identify brodmann areas. Frontiers in Neuroscience, 13, 1159. 10.3389/fnins.2019.01159 31787866 PMC6856631

[R33] Song C, Jeon S, Lee S, Ha HG, Kim J, & Hong J. (2018). Augmented reality-based electrode guidance system for reliable electroencephalography. BioMedical Engineering OnLine, 17, 64. 10.1186/s12938-018-0500-x 29793498 PMC5968572

[R34] Taberna GA, Guarnieri R, & Mantini D. (2019). SPOT3D: Spatial positioning toolbox for head markers using 3D scans. Scientific Reports, 9, 12813. 10.1038/s41598-019-49256-0 31492919 PMC6731320

[R35] Taberna GA, Marino M, Ganzetti M, & Mantini D. (2019). Spatial localization of EEG electrodes using 3D scanning. Journal of Neural Engineering, 16, 026020. 10.1088/1741-2552/aafdd1 30634182

